# Physicochemical and sensorial properties of tomato leathers at different drying conditions

**DOI:** 10.1111/1750-3841.17061

**Published:** 2024-04-05

**Authors:** Eren Basdemir, Alev Emine Ince, Sakine Kizgin, Baris Ozel, Ozlem Ozarda, Servet Gulum Sumnu, Mecit Halil Oztop

**Affiliations:** ^1^ Department of Food Engineering Middle East Technical University Ankara Turkey; ^2^ Food Processing Department Kahramankazan Vocational School, Baskent University Ankara Turkey; ^3^ SELUZ Fragrance and Flavor Company Istanbul Turkey

**Keywords:** drying, NMR relaxometry, sensory analysis, tensile strength, tomato leather

## Abstract

**Abstract:**

Tomato leather as a healthy alternative to traditional fruit leathers was formulated. A tray dryer with changing temperature (50, 60, and 70°C) and relative humidity (5%, 10%, and 20%) was used to achieve the best product in terms of color, water distribution, lycopene content, mechanical, and sensorial properties. Color change was the highest at 70°C due to the Maillard reaction. Lycopene content was also the highest at 70°C. Time domain‐NMR relaxometry showed that water distribution of all samples was homogeneous and similar to each other. Processing conditions affected mechanical properties significantly. The highest tensile strength was observed at 70°C, possibly due to the denatured proteins. Sensory analysis indicated better flavor development at 70°C, whereas overall acceptability of samples was higher at 50°C. The results of this study showed the main processing parameters of tomato leather with a minimal amount of ingredients, with acceptable mechanical and sensorial properties.

**Practical Application:**

Tomato leather was produced by using minimal amount of ingredients. Taste of the leather was found acceptable, as a salty snack food. Therefore, this product can be produced economically and it has a high potential to be consumed as an alternative to conventional fruit leathers.

## 1 INTRODUCTION

Tomato is one of the main components of the Mediterranean diet, and it contains a high amount of lycopene which is associated with a lower risk of cancer and cardiovascular diseases (Farinetti et al., 2017). The high antioxidant content of tomatoes, including carotenoids, ascorbic acid, and polyphenols, is effective in inhibiting the reactions initiated by reactive oxygen species (Grosso et al., 2013). To maximize the health benefits, the production of functional and stable tomato products can be a proper strategy. Processed tomato products, such as ketchup, sauce, and juice, are widely consumed around the world. As the production of tomatoes is abundant in Mediterranean countries including Turkey, the production of processed tomato products is also common (Hepsağ & Kizildeniz, 2021).

Fruit leathers, which are mostly in the form of flexible sheets, are produced to preserve or increase the nutritional value of the fruits (Kurniadi et al., 2022). Fruit leathers are produced by mixing the fruit puree with sugar, pectin, coloring agents, protein, and starch and then by drying into sheet‐like shapes (Diamante et al., 2014). Before the drying step, heat treatment is often applied for inactivating the enzymes and concentrating the pulp (da Silva Simão et al., 2020). Traditional fruit leathers contain a high amount of sugar and therefore have too much calories. Previously, tomato leathers were also prepared with the addition of high amount of sugar to match the taste and texture with traditional fruit leather taste (Fiorentini et al., 2015). However, to the best of our knowledge, there was no study on a salty tomato leather fortified with proteins.

There are many studies on the chemical, physical, textural, and sensorial properties of different fruit leathers, such as apple, pear, strawberry, kiwi, jackfruit, plum, and grape (Bala et al., 2005; Concha‐Meyer et al., 2016; Demarchi et al., 2013; Huang & Hsieh, 2006; Maskan et al., 2002; Nizamlioglu et al., 2022). These studies showed that the physicochemical properties of the fruit leathers, such as antioxidant capacity, color, taste, or softness, depend on the processing conditions including drying temperature and humidity. For instance, high processing temperatures have adverse effects on the physicochemical properties, like color change and denaturation of bioactive compounds. In a previous study, the decrease of antioxidant activity of kiwi leather was reported during processing or storage at high temperatures (Concha‐Meyer et al., 2016). Additionally, as the presence of sugar increases the mechanical properties, such as tensile strength, due to holding sufficient water; changing sugar and pectin concentrations in kiwifruit have been reported to change the mechanical properties (Phimpharian et al., 2011; Vatthanakul et al., 2010).

The drying of leathers is conventionally done under sun, which is the simplest method and the sun‐dried products often have a desired appearance and a gummy texture; however, there are also disadvantages of sun drying including long drying times, exposure to the environmental contamination, and dependency on weather (Diamante et al., 2014; Hazra et al., 2020; Maskan et al., 2002). To avoid these undesirable changes, tunnel or forced air dryers, which are faster, safer, and better controllable, are used (Bala et al., 2005; Chen & Martynenko, 2018; Hazra et al., 2020). Additionally, these methods can be used at any time of the year to meet the customers’ requirement in the industry. During drying, fruit leathers may undergo several physicochemical changes affecting the quality of the final product (Diamante et al., 2014). Such changes include the loss of phenolic content, ascorbic acid, volatiles, taste, and color particularly when prolonged heating is applied during drying (Okilya et al., 2010; Yılmaz et al., 2017). Therefore, depending on the fruit type and the formulation, the correct drying method should be selected.

Tray or belt dryers are commonly used for fruit leather production at an industrial scale. The drying temperature is the most important parameter that affects the quality of the final product. Besides temperature, drying time, slab thickness, airflow speed, and humidity of air also affect the physicochemical and nutritional properties of fruit leathers (da Silva Simão et al., 2020; Maskan et al., 2002). For instance, at high temperatures, color change and loss of antioxidants are the main problems (Chen & Martynenko, 2018). Alternatively, physical problems, such as crack formation on the surface or the sticky structure may arise if the relative humidity (RH) of air is not adjusted (Valenzuela & Aguilera, 2015). As the appearance and texture of fruit leathers are important for consumers, the proper adjustment of the processing parameters is also important.

Although there are some studies on the production of fruit leathers (Diamante et al., 2013, 2014; Huang & Hsieh, 2006), there has been no reported study on the production of a salty tomato leather so far. Tomato leather was formulated in such a way that the number of ingredients was kept minimal and none of the added ingredients would give the consumer the impression of a “caloric product.” As stated before, the goal was to design a new product by using Mediterranean ingredients so that the young population in Mediterranean regions would show a new interest. The main ingredient was the tomato juice. The olive powder, pea protein isolate, and salt were added for a better taste and increasing the functionality of the product. The main criteria for the preparation of the leather samples were chosen as the *a_w_* and moisture content, as these directly influence the shelf life of the product. In this study, the production of a tomato leather at different processing conditions was studied. The two main parameters of the tray dryer, namely, temperature and humidity, were tested. The leathers were analyzed in terms of their physical (*thickness, color, and mechanical properties*), chemical (*water activity, moisture content, and lycopene content*), and sensorial properties. Time domain (TD) NMR relaxometry experiments were also included to have an idea about the water distribution within the samples.

## 2 MATERIALS AND METHODS

### 2.1 Materials

Fresh tomatoes (*Solanum lycopersicum*) were obtained from The Kraft Heinz Food. Pea protein isolate was obtained from Vegrano. Olive powder was prepared using freeze drying process following high‐pressure homogenization of green olives (Argun, 2022). Salt was bought from a local market. Acetone, hexane, ethanol, potassium nitrate, sodium bromide, sodium chloride, sodium hydroxide, magnesium chloride, and potassium acetate were purchased from Merck; potassium chloride and potassium carbonate were purchased from IsoLab.

### 2.2 Preparation of tomato leathers

A hot break procedure was applied to the fresh tomatoes to inactivate the enzymes, particularly polygalacturonase and pectin methyl esterase (Goodman et al., 2002). For this, first, tomatoes were washed and peeled manually. Then, heat was applied (85°C for 3 min) together with mixing by using Vorwerk Thermomix^®^ TM5. After this treatment, the obtained tomato juice was cooled and placed into the freezer (Arçelik) at −18°C for further use.

For the preparation of leathers, frozen tomato juice was thawed by keeping it at 4°C for 1 day. Then, the juice was filtered through a 0.2 mm sieve to remove seeds and remaining skins. The main ingredient of leather was the tomato juice with an average °Brix of 5.2 and pH of 4.2. Olive powder, pea protein isolate, and salt were added at a ratio of 0.5% (w/w) each. This mixture was pre‐homogenized by using a rotor‐stator homogenizer (Ultra Turrax^®^ T25, IKA WERKE) at 13,800 rpm for 2 min, and then a high‐pressure homogenizer (GEA PandaPlus) was used for 2 passes at 500 bars to ensure well integration of the pea protein into the matrix.

### 2.3 Processing conditions of the tray dryer

**FIGURE 1:**
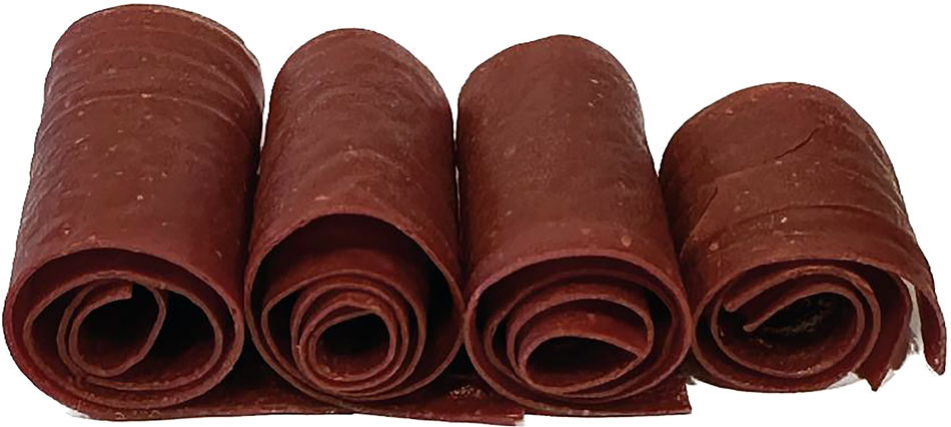
Picture of leather samples.

Two hundred grams of this mixture were poured onto a baking paper inside a specially designed metal frame of 12 × 20 × 0.7 cm^3^ (Figure 1). Then, the sample was dried in a tray dryer. Drying procedure was carried out using a tray dryer (Eksis) operated at 50, 60, and 70°C with 5%, 10%, and 20% RH. Air speed was set to 1 m/s, and the rotation speed of the trays was adjusted to 6 rpm. Drying time varied according to the temperature and RH conditions. Duration of drying was decided based on the water activity (*a_w_*) values. Leathers were dried until they had an *a_w_* of 0.4. Therefore, the drying time changed from 271 to 515 min.

### 2.4 Analysis of the tomato leathers

#### 2.4.1 Thickness measurement

The thickness of the leathers was measured by using a digital vernier caliper (Max‐Extra). The average thickness of six different points of one leather was reported.

#### 2.4.2 Color measurement

A portable spectrocolorimeter (Serlab SL400) was used to determine the L^*^, a^*^, and b^*^ values of the leathers. The Δ*E* values were calculated using the following formula:

(1) $$\Delta E={\left({\left(L*-L\right)}^{2}+{\left(a*-a\right)}^{2}+{\left(b*-b\right)}^{2}\right)}^{1/2},$$

where the *L* ^*^, *a* ^*^, and *b* ^*^ values were for the leather samples, and *L, a*, and *b* values were for a white paper, as reference.

Results were obtained from three different points of one sample, and the average values were reported.

#### 2.4.3 Water activity and moisture content measurements

A water activity meter (Aqua Lab 4TE, Decagon Devices Inc.) was used at 25°C to detect the *a_w_* of leather samples.

Moisture content was determined gravimetrically. Samples were kept in an oven (Mikrotest) at 105°C for 3 h. Moisture content was calculated in wet basis by taking the weight difference between fresh and dried samples:

(2) $$\mathrm{Moisture}\;\mathrm{content}\;\left(\%\right)=\left(W_{2}-W_{3}\right)\times 100/\left(W_{2}-W_{1}\right),$$

where *W*_1_ is the weight (g) of the empty dish, *W*_2_ is the weight (g) of the dish with wet sample, and *W*_3_ is the weight (g) of the dish with dried sample.

#### 2.4.4 Lycopene content measurement

Conventional solvent extraction is a commonly used method in order to quantify the lycopene in tomatoes and its powdered forms (Shi & Maguer, 2000). One gram of leather was put into a 50 mL falcon tube with 10 mL of distilled water and subsequently shaken by a digital orbital shaker (Daihan Scientific) at 200 rpm for 24 h to dissolve the sample. Then, 25 mL of hexane:acetone:ethanol (2:1:1) mixture was added to the dissolved the sample for lycopene extraction.

Although the primary solvent used in lycopene extraction was hexane, there was a synergistic effect when hexane was mixed with acetone and ethanol (Periago et al., 2004). The mixture was vortexed for 2 min and then kept in the dark for 10 min to obtain the upper hexane phase and prevent exposure to light.

The spectrophotometric method was used to determine the exact amount of lycopene in tomato leather. The nonpolar supernatant phase containing hexane and lycopene was transferred to a quartz cuvette (10 mm path‐length), and the absorbance value was determined with a spectrophotometer (Optizen POP UV–Visible, Mecasys) at 503 nm. Hexane was used as the blank. The amount of lycopene was calculated by using the following equation (Aliyu et al., 2020):

(3) $$\mathrm{lycopene}\;\left(\mathrm{mg}/\mathrm{kg}\;\mathrm{fresh}\;\mathrm{wt}.\right)=A_{503}\times 171.7/W,$$

where *W* is the weight of the sample (g), and *A* is the absorbance at 503 nm. 171.7 mM^−1^ is the extinction coefficient for lycopene in hexane (Zechmeistbr et al., 1943).

#### 2.4.5 Mechanical properties

Brookfield CT3 model texture analyzer (Middleboro) was used for the determination of the mechanical properties of tomato leather. Tensile strength test was applied on 10 mm wide and 100 mm long leather strips with characteristic thickness using a TA‐DGA fixture. The strips were fastened between the fixture clamps 90 mm apart. The sample was pulled up to reach the 30 mm target distance at 0.50 N trigger load, and 0.50 mm/s test speed until breaking off. Tensile strength (N), elongation at break (mm), and fracture work (g cm) were determined. The tensile strength is the maximum stress that a leather sample can sustain; therefore, it expressed the mechanical resistance (Nandane & Jain, 2014). Elongation at break indicates the plasticity of the sample (Chakravartula et al., 2019). The fracture work is the energy required to propagate a material crack. Measurements were done in three replicates, and the average values were reported.

#### 2.4.6 NMR relaxometry

The relaxation times (*T*_2_) were measured with an NMR system (Pure Devices GmbH) operating at ^1^H frequency of 22.40 MHz. For *T*_2_ measurements, Carr–Purcell–Meiboom–Gill (CPMG) pulse sequence was used. CPMG parameters were set as; repetition time of 1000 ms, echo time of 2 ms, 20 acquisition points, and 4 scans.

#### 2.4.7 Sorption isotherm of leather samples

Sorption isotherm of tomato leathers was determined by using the static method (Erbas et al., 2016). In this method, leather samples were kept in tightly closed jars containing different saturated salt solutions at 25°C (Table 1). Experimental data fitted well with the Guggenheim–Anderson–de Boer (GAB) model with an *R* ^2^ value of 0.98. The GAB equation is expressed as

(4) $$\mathrm{MC}=\frac{M_{0}\mathrm{CK}a_{w}}{\left(1-Ka_{w}\right)\times \left(1-Ka_{w}+\mathrm{CK}a_{w}\right)},$$

where *M*_0_ is the monolayer *MC*, and *C* and *K* are the free sorption constants (Sahin & Sumnu, 2006). The obtained GAB equation for the leather samples was as follows:

(5) $$\frac{a_{w}}{MC}=-0.0601\;{a_{w}}^{2}+0.0711\;a_{w}+0.0001.$$

**TABLE 1:** Water activity values of saturated salt solutions used in the sorption isotherm determination of tomato leather at 25°C (Sahin & Sumnu, 2006).

Solution	*a_w_* at 25°C
Sodium hydroxide	0.082
Potassium acetate	0.225
Magnesium chloride	0.328
Potassium carbonate	0.432
Sodium bromide	0.576
Sodium chloride	0.753
Potassium chloride	0.843
Potassium nitrate	0.936

#### 2.4.8 Scanning electron microscopy

To investigate the surface morphology of tomato leathers, SEM analysis was performed by the METU Central Laboratory. Fresh samples were coated with Au‐Pd with a thickness of 3 nm before the imaging. Then, the samples were analyzed by using an electron microscope (Quanta 400F FESEM) at different magnifications.

### 2.5 Sensory analysis

Tomato leather samples were evaluated with a team of 5 trained panelists. The samples were evaluated considering the taste, odor, color, and texture parameters. Flavor profile analysis methodology (ISO 6564:1985) was used. The *flavor profile* describes flavor in terms of five major components: character attributes, attribute intensity, order of attribute appearance, aftertaste, and amplitude (*the overall impression of the flavor components that can and cannot be analyzed*). The original scale for the flavor profile was five points: *not present, threshold, slight, moderate, and strong*.

### 2.6 Statistical analysis

The numerical results obtained from various experiments were analyzed by two‐way analysis of variance by using Minitab 21 (Minitab Inc.). The groups are identified whether the means of the data is significantly different or not using Tukey's test at the 95% confidence interval. Correlations were performed by using Pearson's correlation test.

## 3 RESULTS AND DISCUSSION

### 3.1 Physicochemical properties

#### 3.1.1 Moisture content and *a_w_*

Fruit leathers often have a soft chewy texture due to high moisture content. However, to prevent microbial growth, the *a_w_* should be low enough, such as below 0.6 (Majumdar et al., 2018). Therefore, the tomato leathers were dried to a moisture content of 25%, which corresponded to an *a_w_* of around 0.4 (Table 2). Previous studies on fruit leathers, such as apple and blackcurrant, reported that the moisture content values were between 12% and 27% and the *a_w_* ranged from 0.4 to 0.7 (Diamante et al., 2013, 2014). Similarly, the moisture content and *a_w_* of tomato leathers were between these values. However, the main difference between other fruit leathers and tomato leathers was the amount of sugar. In some of the tomato leather studies, sugar was added to the formulation for the taste and textural properties (Chhetri et al., 2022). The presence of sugar in different fruit leathers resulted in a higher moisture content, which gives a chewy texture to the leather (Diamante et al., 2013). On the other hand, in our study, the tomato leather contained no additional sugar; therefore, the efficient absorption of water in the bulk was low. The sugar that was inherently present in the tomato held water, and this amount of sugar was too low to give a chewy structure. Therefore, at the desired chewy texture of tomato leathers, the water activity and the moisture content became higher than the other fruit leathers, which contain high fructose sugar, such as grapes.

**TABLE 2:** Moisture content, water activity, and lycopene concentration of tomato leather samples processed at different drying conditions.

	Drying conditions					
Sample	*T* (°C)	RH (%)	Drying time (min)	Moisture content (%)	Water activity (*a_w_*)	Lycopene (mg/kg DM)
**S1**	50	5	450	23.93 ± 0.31^bc^	0.38 ± 0.01^a^	396.1 ± 1.1^e^
**S2**	50	10	478	23.16 ± 0.40^cd^	0.39 ± 0.05^a^	431.1 ± 7.9^e^
**S3**	50	20	518	27.14 ± 0.31^a^	0.38 ± 0.01^a^	193.1 ± 1.5^g^
**S4**	60	5	373	25.34 ± 0.42^ab^	0.37 ± 0.01^a^	321.0 ± 9.1^f^
**S5**	60	10	391	25.03 ± 0.49^b^	0.43 ± 0.00^a^	577.6 ± 12.0^d^
**S6**	60	20	396	22.00 ± 0.28^de^	0.42 ± 0.03^a^	278.8 ± 5.9^f^
**S7**	70	5	283	27.07 ± 0.73^a^	0.45 ± 0.05^a^	937.7 ± 23.1^a^
**S8**	70	10	321	20.92 ± 0.60^e^	0.42 ± 0.04^a^	858.4 ± 23.5^b^
**S9**	70	20	332	20.51 ± 0.40^e^	0.39 ± 0.01^a^	647.6 ± 6.5^c^

*Note*: Different letters indicate significant differences (*p* < 0.05) within the samples. Errors are represented as standard deviations.

Abbreviation: RH, relative humidity.

The different process conditions led to different drying times for reaching the *a_w_* value of 0.4 (Table 2). The drying times of samples decreased with the increasing dryer temperature from 50 to 70°C, and with decreased RH of air from 20% to 5% (Table 2). As expected, the high temperature and low humidity in the dryer yielded faster drying. However, fast drying sometimes created structural and functional problems in leathers, such as low sensory attributes and decreased vitamin content (Diamante et al., 2013). In our preliminary experiments, we confronted with cracked structures in tomato leathers upon fast drying. For instance, at high drying temperatures, keeping the color of the leather was a challenge. Therefore, mild drying conditions could be more preferable for better sensorial and functional properties.

#### 3.1.2 Lycopene content

An important functional component of the tomatoes is lycopene. The lycopene content of leathers is presented in Table 2. The highest concentration was observed for samples dried at 70°C. The availability of lycopene is known to increase through treatments such as heating and high‐pressure homogenization, as the cell membranes are broken and carotenoids can come out of the cell (D'Evoli et al., 2013; Mert, 2012). Although the availability of lycopene was reported to increase with increasing temperatures, particularly above 100°C (Colle et al., 2010), for tomato leathers, a similar trend from 50 to 70°C has been shown. The temperature was found to be statistically significant (*p* < 0.05) on the lycopene content. On the other hand, the other processing parameter, humidity was found insignificant on the lycopene content of tomato leathers (*p* > 0.05).

#### 3.1.3 Moisture sorption behavior

**FIGURE 2:**
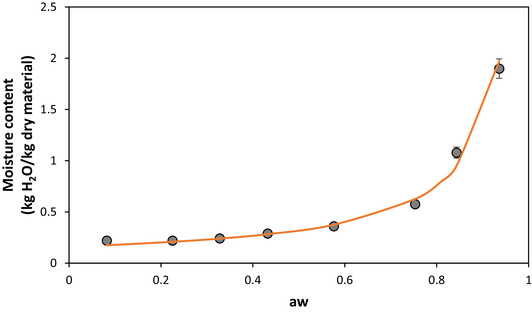
Moisture sorption isotherm of tomato leather at 25°C. Solid line represents the Guggenheim–Anderson–de Boer (GAB) model.

A sorption isotherm is a highly useful tool in the course of processing, packaging, and storing of foods and defines the physical, chemical, and biochemical relation between water and food (Erbas et al., 2016). One can gain insight for the moisture distribution and migration by using the sorption isotherm. Moisture sorption isotherm of the tomato leather was obtained at 25°C and is shown in Figure 2. Experimental data showed the best fit with the GAB model. Previously, grape leathers were also reported to show the best fit with the GAB model (Kaya et al., 2002). The estimated constants *M*_0_, *K*, and *C* for tomato leather were found to be 14.03, 0.844, and 844, respectively. The value of *C* was larger than 2; therefore, the model had a Type 2, that is, sigmoidal shape curve (Blahovec & Yanniotis, 2008). Type 2 isotherms were reported to represent the typical behavior for intermediate moisture content products (Al‐Muhtaseb et al., 2010). In these types of isotherms, at low *a_w_* s, the adsorption monolayer was constituted by the saturation of polar and hydrophilic components with water molecules. As the *a_w_* increased, the additional water molecules formed the multilayer coverage, and water molecules are accumulated in intermolecular free spaces.

Another feature of the newly designed food product is its color. Color has great importance for most food products due to its considerable influence on consumer preference (Rathee & Rajain, 2019). Therefore, the effect of different drying conditions on the color of samples was investigated.

#### 3.1.4 Color properties

L^*^, a^*^, b^*^, and Δ*E* values of samples are shown in Table 3. These values showed slight differences depending on the processing conditions (*p* < 0.05). Particularly, L^*^ and a^*^ values correlated well with the browning of samples, which was also previously stated by others (Nayaka et al., 2022). The increase in a^*^ values could be related to both browning and the availability of lycopene at increasing temperatures. This finding is also in line with the previous research stating that the increase of a^*^ values for grape leather samples with increasing drying temperatures (Maskan et al., 2002).

**TABLE 3:** The effect of drying temperature and humidity on L^*^, a^*^, and b^*^ values of the tomato leathers.

Sample (conditions)	L^*^ (brightness/dark)	a^*^ (red/green)	b^*^ (yellow/blue)	Δ*E*
S1 (50°C, 5% RH)	31.2 ± 0.8^b^	19.7 ± 0.1^b^	10.7 ± 0.3^b^	38.4 ± 0.8^b^
S2 (50°C, 10% RH)	32.4 ± 1.1^ab^	19.2 ± 1.8^b^	10.7 ± 1.3^b^	39.1 ± 2.1^b^
S3 (50°C, 20% RH)	34.4 ± 1.0^ab^	22.3 ± 0.6^ab^	13.1 ± 0.6^ab^	43.0 ± 1.3^ab^
S4 (60°C, 5% RH)	33.0 ± 1.1^ab^	21.8 ± 0.8^ab^	11.4 ± 0.4^b^	41.1 ± 1.2^ab^
S5 (60°C, 10% RH)	32.5 ± 0.4^ab^	22.5 ± 1.0^ab^	12.1 ± 0.1^ab^	41.3 ± 0.8^ab^
S6 (60°C, 20% RH)	34.6 ± 1.2^ab^	24.1 ± 2.3^ab^	14.3 ± 1.5^ab^	44.5 ± 2.6^an^
S7 (70°C, 5% RH)	32.8 ± 0.1^ab^	19.5 ± 0.4^b^	11.0 ± 0.1^b^	39.6 ± 0.2^b^
S8 (70°C, 10% RH)	35.9 ± 1.0^a^	26.8 ± 2.5^a^	15.6 ± 1.9^a^	47.4 ± 2.8^a^
S9 (70°C, 20% RH)	33.4 ± 0.8^ab^	21.6 ± 0.1^ab^	12.8 ± 0.8^ab^	41.7 ± 0.8^ab^

*Note*: Different letters indicate significant differences (*p* < 0.05) within the samples. Errors are represented as standard deviations.

Abbreviation: RH, relative humidity.

In fresh tomatoes, color change may be due to the oxidation of phenolic compounds because of the enzymatic activity, particularly the action of polyphenol oxidase (PPO) (Spagna et al., 2005). PPO, giving resistance against microorganisms and insects, is inherently present in tomatoes, and its activity is the highest at pH 4.8 and 40°C (Kampatsikas et al., 2019; Spagna et al., 2005). During the hot break process, the PPO was inactivated, as the tomato juice was heated up to 85°C. Therefore, we assume that the PPO did not take part in the color change of tomato leathers. For the tomato leathers, the slight changes in L^*^ and a^*^ values indicated the occurrence of Maillard reactions to a certain extent (Maskan et al., 2002). The presence of protein in the formulation and the reducing sugars inherently present in tomatoes should have resulted in browning reactions with the effect of heating.

#### 3.1.5 SEM images

**FIGURE 3:**
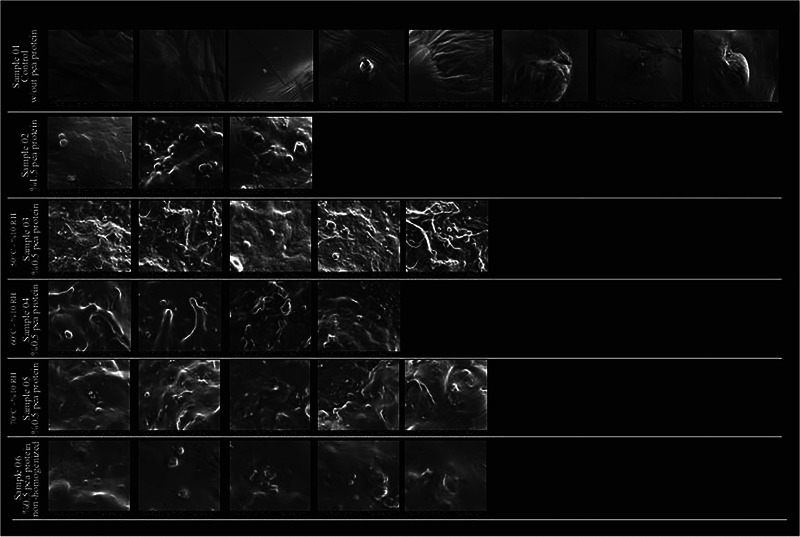
SEM pictures of tomato leathers prepared at different conditions.

The SEM pictures of different tomato leathers are shown in Figure 3. From these pictures, the surface inhomogeneity in the microstructure of samples in the presence of protein can be deduced as the control sample (first line) was smoother and included more fibril‐like structures. The non‐homogenized sample (last line) seemed to have larger, spherical aggregates, possibly due to the presence of protein, than the homogenized samples. Other samples except the control had certain roughness on their surfaces. The sample dried at 50°C appeared to have a rougher surface than the samples dried at higher temperatures. The reason of this roughness could be the aggregation of pea protein isolate during the pressure‐homogenization step, the presence of olive powder, or both. On the other hand, pea proteins may have also attained more extended conformations at 70°C due to partial denaturation at this higher temperature. It is obvious from the pictures that the homogenization step and the presence of protein had a significant effect on the structure.

#### 3.1.6 NMR relaxometry

Water distribution within the samples was analyzed by transverse relaxation experiments. Relaxation spectrum analysis indicated that the relaxation behavior was bi‐exponential. Therefore, the relaxation peak times and respective normalized peak area values are reported in Table 4. The first peak time (*T*_21_) values changed between 42 and 53 ms but did not show any statistical change. The second peak time values were in the range of 165.5 and 195 ms, but again all the data were significantly the same. Moreover, relative peak areas also demonstrated stable profiles with no significant change among the different drying parameters. This indicates that leather samples possessed similar water distribution properties at different drying conditions. Leather formulations showed a strong stability at changing temperatures and RH conditions in terms of consistency of water distribution. Thus, the temperature and RH range studied in this study did not significantly change the *T*_2_ regime of the leather samples. Additionally, the distribution of relative peak areas of the respective peak times also suggested a homogeneous water distribution within the samples, as the area values were close to each other. Generally, a lower peak time is associated with restricted water mobility that is in bound‐state, whereas a higher value is attributed to the water fraction with a higher mobility (Mariette, 2009). However, the proximity of the two distinct relaxation peak areas showed that there was not a major distinction between the natures of the transverse relaxation in both fractions. The main reason could be the lack of a distinct free‐water population providing longer relaxation (Hashemi et al., 2010). This trend was also in agreement with the similar *a_w_* values of the samples at different drying conditions as demonstrated in Table 2. Although moisture contents of the samples showed some variations with respect to the changing drying parameters, the amount of water present within the samples did not determine the nature of water distribution. As *T*_2_ depends on the dephasing of neighboring spins, interactions between the water phase and the surrounding polymer network determined the homogeneity of the water distribution within the leather samples (Ozel & Oztop, 2021). Consequently, stable *T*_2_ profiles of the samples demonstrated that the drying process conditions used in this study resulted in similar and relatively homogeneous drying of the leathers.

**TABLE 4:** *T*_2_ and respective area values in bi‐exponential mode for tomato leathers produced at different conditions.

Sample	*T*_21_	*A*_21_	*T*_22_	*A*_22_
S1 (50°C, 5% RH)	45.0 ± 1.4^a^	55.6 ± 2.6^ab^	166.5 ± 6.4^a^	44.5 ± 2.6^ab^
S2 (50°C, 10% RH)	46.0 ± 0.0^a^	55.6 ± 0.6^ab^	170.0 ± 0.0^a^	44.5 ± 0.6^ab^
S3 (50°C, 20% RH)	49.0 ± 4.2^a^	56.4 ± 1.0^ab^	172.0 ± 0.0^a^	43.6 ± 1.0^ab^
S4 (60°C, 5% RH)	48.5 ± 3.5^a^	59.6 ± 2.3^ab^	178.5 ± 10.6^a^	40.5 ± 2.3^ab^
S5 (60°C, 10% RH)	53.0 ± 5.7^a^	67.5 ± 4.1^a^	195.0 ± 25.5^a^	32.5 ± 4.1^b^
S6 (60°C, 20% RH)	51.5 ± 2.1^a^	60.2 ± 3.0^ab^	185.5 ± 9.2^a^	39.8 ± 3.0^ab^
S7 (70°C, 5% RH)	42.0 ± 1.4^a^	53.0 ± 1.6^b^	165.5 ± 3.5^a^	47.1 ± 1.6^a^
S8 (70°C, 10% RH)	48.0 ± 2.8^a^	58.9 ± 5.4^ab^	183.5 ± 23.3^a^	41.1 ± 5.4^ab^
S9 (70°C, 20% RH)	53.0 ± 8.5^a^	61.6 ± 6.3^ab^	188.5 ± 19.1^a^	38.5 ± 6.3^ab^

*Note*: Different letters indicate significant differences (*p* < 0.05) within the samples. Errors are represented as standard deviations.

Abbreviation: RH, relative humidity.

### 3.2 Mechanical properties

Mechanical properties allow for determining the strength of leather samples when they are processed, packed, or handled to keep their integrity (Valenzuela & Aguilera, 2013). These properties depend on several factors such as moisture content and thickness, which also depend on processing parameters. The average moisture content of the samples was already reported as 25%, and the thickness of leathers varied from 0.61 to 0.71 mm (Table 5).

**TABLE 5:** Effect of drying conditions on the mechanical properties of tomato leathers.

Sample No	Thickness (mm)	Tensile strength (N)	Elongation at break (mm)	Fracture work (g cm)
S1 (50°C, 5% RH)	0.61 ± 0.03^b^	8.48 ± 0.50^a^	16.31 ± 0.49^e^	1445 ± 21^bc^
S2 (50°C, 10% RH)	0.62 ± 0.04^b^	6.22 ± 0.35^bcd^	18.33 ± 0.13^cd^	1447 ± 112^bc^
S3 (50°C, 20% RH)	0.65 ± 0.01^ab^	3.66 ± 0.28^e^	18.65 ± 0.23^bc^	735 ± 39^d^
S4 (60°C, 5% RH)	0.71 ± 0.04^a^	7.51 ± 0.73^ab^	16.66 ± 0.06^de^	1307 ± 81^c^
S5 (60°C, 10% RH)	0.67 ± 0.01^ab^	8.31 ± 0.90^a^	19.42 ± 0.35^bc^	1669 ± 139^ab^
S6 (60°C, 20% RH)	0.71 ± 0^a^	7.37 ± 0.76^abc^	21.84 ± 0.73^a^	1748 ± 77^a^
S7 (70°C, 5% RH)	0.70 ± 0.01^a^	8.02 ± 0.32^a^	19.41 ± 0.01^bc^	1724 ± 41^ab^
S8 (70°C, 10% RH)	0.65 ± 0.01^ab^	5.44 ± 0.50^d^	18.23 ± 0.97^cd^	1154 ± 4^c^
S9 (70°C, 20% RH)	0.68 ± 0.01^ab^	5.98 ± 0.30^cd^	20.44 ± 0.06^ab^	1323 ± 58^c^

*Note*: Different letters indicate significant differences (*p* < 0.05) within the samples. Errors are represented as standard deviations.

Abbreviation: RH, relative humidity.

In Table 5, the mechanical properties of leather samples are also reported as tensile strength, elongation at break, and fracture work. The tensile strength values ranged from 3.66 to 8.48 N. The highest tensile strength values were observed in the samples dried at 5% or 10% RH, which had a significant effect (*p* < 0.05). Fracture work, that is, the energy for rupture, for the leather samples had a similar trend with the tensile strength. Drying of leathers at 60°C yielded the highest tensile strength, which could be related to the difference in moisture content and the thickness of samples (Valenzuela & Aguilera, 2013). In addition, at increasing drying temperatures, the increase in tensile strength may be expected as a result of the partial protein denaturation and network formation. Although the denaturation temperature of pea proteins is above 70°C (Mession et al., 2015), the interactions between proteins or protein and pectin may have occurred upon heating, which resulted in the network formation. The network formation at different cross‐linking densities would have taken place (Fernández‐Pan et al., 2010), and therefore, the mechanical properties changed. Alternatively, the pea protein isolate could form a complex with pectin due to the Maillard reaction at increasing temperatures (Bonnaillie et al., 2014).

For elongation at break, both temperature and humidity were found to be significant (*p* < 0.05). Increasing temperature and humidity during the process generally increased the elongation values, which indicated an increasing elasticity. The highest elongation values were obtained at drying conditions with 20% humidity for all temperatures. At constant thickness, elongation at break increased with increasing humidity in leather samples. Tomato leather samples can be compared with the edible films in literature. In a previous study, casein‐based films were prepared at different RHs and when the humidity increased, tensile strength was found to decrease, whereas elongation at break was found to increase (Bonnaillie et al., 2014), which is also in line with the results of tomato leathers.

The mechanical properties of tomato leathers were found to be dependent on both formulation and processing conditions, which was also stated by others (Valenzuela & Aguilera, 2013). The protein and pectin in the formulation modified the textural properties by forming a network. In addition, the drying temperature and humidity affected the mechanical properties by altering the extent of network formation.

### 3.3 Sensory evaluation

Tomato leather samples were evaluated considering the taste, odor, color, and texture parameters. In general, panelists considered the color of all samples preferable. However, the texture of all samples was found to be sticky, as the samples possessed high resistance to chewing. This was mainly due to the less amount of glucose and fructose compared to the other fruit leathers. The amount of sugar inherently present in tomatoes was ∼2.5%, whereas high‐fructose‐containing fruits contain ∼16% total sugar (Roha et al., 2013), which directly affected the textural and sensorial properties. As the sugar molecules can hold water in the structure, they are able to increase the moisture content of the leather, thereby giving a chewy and soft structure (Diamante et al., 2013). Therefore, the dry and sticky texture of tomato leathers was a result of the low moisture content and *a_w_*, which was influenced by the sugar amount. The low *a_w_* of the leathers and the resulting dry texture were consistent with the NMR relaxometry results. *T*_2_ relaxation analysis previously showed that the water distribution was homogeneous for all samples during drying and a free‐water population that would cause high *a_w_* values was missing. Presence of such a distinct free‐water population would lead to a softer product, but this was not the case for the current samples. Therefore, the *T*_2_ relaxation and distribution properties of the leather samples are in agreement with the final dry/sticky texture.

In previous studies, fruit leathers with high sugar content were often found to be preferable (Phimpharian et al., 2011). As a healthier alternative, in tomato leather, the absence of additional sugar created some sensory problems, particularly in its texture. The chewy characteristic was much less compared to the other high‐fructose‐containing fruits.

In the flavor profile analysis, sweetness, sourness, saltiness, tomato paste taste, dry tomato taste, dry fruity notes, rotten notes, green notes, spicy notes, cooked tomato paste, and overall acceptance were reported. These attributes are shown in Table 6. Tomato paste notes were perceived as high in all samples, which can be taken as a positive attribute because in many tomato products, this taste is desirable. In addition, the sourness was felt quite dominant in some samples. High sourness perception could be related to the combination of the original pH of the tomato and the dryness of the product. Although saltiness perception was balanced in most samples, it increased for the samples dried at 70°C, which could be related to the moisture content. As the moisture content of the samples decreased, the saltiness perception was expected to increase. Alternatively, drying at 70°C could enhance the saltiness perception by masking some other dominant flavors. Similarly, the dry tomato taste was found to increase at increasing temperatures. Overall acceptance of samples dried at 60 and 70°C with 20% RH was determined to be high, whereas that of samples dried at 60°C with 5% and 10% RH was low. These results showed that high temperature and high moisture content for drying conditions were better. In a previous study on the sensory properties of plum fruit leathers prepared at 60, 70, and 80°C, the highest results were reported for the ones prepared at 70°C (Sonkar et al., 2019), which is similar to our findings. High drying temperatures could better enhance flavor development. However, interestingly, all the leathers dried at 50°C were also found to be good in terms of overall acceptance. These findings indicate that mild drying conditions increase the overall acceptance of leather samples.

**TABLE 6:** Flavor profile analysis of leather samples produced at different conditions.

Samples attributes	S1 (50°C, 5% RH)	S2 (50°C, 10% RH)	S3 (50°C, 20% RH)	S4 (60°C, 5% RH)	S5 (60°C, 10% RH)	S6 (60°C, 20% RH)	S7 (70°C, 5% RH)	S8 (70°C, 10% RH)	S9 (70°C, 20% RH)
**Sweetness**	1.5	1.5	3.5	3	1	3	1	3	1.5
**Sourness**	2.5	2	2	2	3	3	2.5	3	3
**Saltiness**	2	2.5	2	1.5	2	1	4	4	2.5
**Tomato paste taste**	3	4	3	3.5	2	3	3	3.5	4
**Dry tomato taste**	1	0	0	1	1	1	0	2	3
**Dry fruity notes**	0	0	0	1	1	1	0	0	0
**Rotten notes**	0	0.5	0	2	0	1	0	0	0
**Green notes**	1	0	0	0	0	0	0	0	0
**Spicy notes**	0.5	0	0	0	0	0	0	0	0
**Cooked tomato taste**	0.5	0	0	0	0	0	0	0	0
**Overall acceptance**	3	3	3	1	1.5	3.5	2.5	2	4

Abbreviation: RH, relative humidity.

In conclusion, the drying temperature and RH affected the sensory attributes of tomato leathers. In many fruit leathers, consumers often prefer a stronger taste, texture, and bright, shiny colors.

## 4 CONCLUSION

In this study, a functional tomato leather product with a minimum amount of ingredients and long shelf life was produced for the first time. The temperature and RH of the tray dryer were changed to investigate the physical, chemical, and textural properties of the leathers. The moisture content and *a_w_* values of leathers were optimized considering the textural properties and possible microbial growth. Textural properties were also affected by temperature and RH, possibly due to the partial denaturation and network formation of the protein. According to the TD NMR relaxometry analysis, the drying conditions used provided similar and homogeneous water distribution within the samples as the bi‐exponential relaxation peak time and area values maintained a stable profile. Lycopene content of leathers increased with increasing drying temperatures, which also increased the browning of samples according to the color measurements. Sensorial properties of leathers still needed to be optimized, as the chewiness and stickiness were found to be high for fruit leather. The fruit leathers were sweet; however, the tomato leather was salty. Therefore, a comparison of the two in terms of sensorial properties was difficult. At high temperatures, the flavor development was high for the tomato leathers. However, interestingly, at low drying temperatures, the overall acceptance was high, possibly due to the preservation of color. Tomato leathers can be consumed as snack food between meals, and it is also possible to use them inside sandwiches or as a wrapping material for the formulation of different snack foods. Therefore, further studies may include the hydration and solubility properties of tomato leathers at different conditions such as different pH and ionic strength environments.

## AUTHOR CONTRIBUTIONS

**Eren Basdemir**: Data curation; investigation; formal analysis. **Alev Emine Ince**: Writing—original draft; methodology; supervision. **Sakine Kizgin**: Data curation. **Baris Ozel**: Writing—original draft; writing—review and editing. **Ozlem Ozarda**: Methodology. **Servet Gulum Sumnu**: Conceptualization; writing—review and editing. **Mecit Halil Oztop**: Conceptualization; investigation; funding acquisition; writing—review and editing; project administration; supervision.

## CONFLICT OF INTEREST STATEMENT

The authors declare no conflicts of interest.

## References

[jfds17061-bib-0001] Aliyu, A. , Kabiruyunusa, A. , & Abdullahi, N. (2020). Kinetics of the thermal degradation of lycopene in tomatoes. Croatian Journal of Food Science and Technology, 12(1), 84–89. 10.17508/CJFST.2020.12.1.11

[jfds17061-bib-0002] Al‐Muhtaseb, A. H. , Hararah, M. A. , Megahey, E. K. , McMinn, W. A. M. , & Magee, T. R. A. (2010). Moisture adsorption isotherms of microwave‐baked Madeira cake. LWT—Food Science and Technology, 43(7), 1042–1049. 10.1016/J.LWT.2010.01.003

[jfds17061-bib-0003] Argun, S. (2022). Production and characterization of microfluidized olive powder. Middle East Technical University.

[jfds17061-bib-0004] Bala, B. K. , Ashraf, M. A. , Uddin, M. A. , & Janjai, S. (2005). Experimental and neural network prediction of the performance of a solar tunnel drier for drying jackfruit bulbs and leather. Journal of Food Process Engineering, 28, 552–566.

[jfds17061-bib-0005] Blahovec, J. , & Yanniotis, S. (2008). Gab generalized equation for sorption phenomena. Food and Bioprocess Technology, 1(1), 82–90. 10.1007/s11947-007-0012-3

[jfds17061-bib-0006] Bonnaillie, L. , Zhang, H. , Akkurt, S. , Yam, K. , & Tomasula, P. (2014). Casein films: The effects of formulation, environmental conditions and the addition of citric pectin on the structure and mechanical properties. Polymers, 6(7), 2018–2036. 10.3390/polym6072018

[jfds17061-bib-0007] Chakravartula, S. S. N. , Soccio, M. , Lotti, N. , Balestra, F. , Dalla Rosa, M. , & Siracusa, V. (2019). Characterization of composite edible films based on pectin/alginate/whey protein concentrate. Materials, 12(15), 2454. 10.3390/ma12152454 31374873 PMC6696009

[jfds17061-bib-0008] Chen, Y. , & Martynenko, A. (2018). Combination of hydrothermodynamic (HTD) processing and different drying methods for natural blueberry leather. LWT, 87, 470–477. 10.1016/j.lwt.2017.09.030

[jfds17061-bib-0009] Chhetri, A. J. , Dangal, A. , Shah, R. , Timsina, P. , & Bohara, E. (2022). Nutritional and sensory quality of prepared tomato (*Solanum lycopersicum*) leather. Analytical Science &Technology, 35(4), 169–180.

[jfds17061-bib-0010] Colle, I. J. P. , Lemmens, L. , Tolesa, G. N. , Van Buggenhout, S. , De Vleeschouwer, K. , Van Loey, A. M. , & Hendrickx, M. E. (2010). Lycopene degradation and isomerization kinetics during thermal processing of an olive oil/tomato emulsion. Journal of Agricultural and Food Chemistry, 58(24), 12784–12789. 10.1021/jf102934u 21080712

[jfds17061-bib-0011] Concha‐Meyer, A. A. , D'Ingnoti, V. , Saez, B. , Diaz, R. I. , & Torres, C. A. (2016). Effect of storage on the physico‐chemical and antioxidant properties of strawberry and kiwi leathers. Journal of Food Science, 81(3), C569–C577. 26799705 10.1111/1750-3841.13214

[jfds17061-bib-0012] da Silva Simão, R. , de Moraes, J. O. , Carciofi, B. A. M. , & Laurindo, J. B. (2020). Recent advances in the production of fruit leathers. Food Engineering Reviews, 12(1), 68–82. 10.1007/s12393-019-09200-4

[jfds17061-bib-0013] Demarchi, S. M. , Quintero Ruiz, N. A. , Concellón, A. , & Giner, S. A. (2013). Effect of temperature on hot‐air drying rate and on retention of antioxidant capacity in apple leathers. Food and Bioproducts Processing, 91(4), 310–318. 10.1016/J.FBP.2012.11.008

[jfds17061-bib-0014] D'Evoli, L. , Lombardi‐Boccia, G. , & Lucarini, M. (2013). Influence of heat treatments on carotenoid content of cherry tomatoes. Foods, 2(3), 352–363. 10.3390/foods2030352 28239121 PMC5302297

[jfds17061-bib-0015] Diamante, L. , Li, S. , Xu, Q. , & Busch, J. (2013). Effects of apple juice concentrate, blackcurrant concentrate and pectin levels on selected qualities of apple‐blackcurrant fruit leather. Foods, 2(3), 430–443. 10.3390/foods2030430 28239127 PMC5302290

[jfds17061-bib-0016] Diamante, L. M. , Bai, X. , & Busch, J. (2014). Fruit leathers: Method of preparation and effect of different conditions on qualities. International Journal of Food Science, 2014, 1–12. 10.1155/2014/139890 PMC4745556 26904618

[jfds17061-bib-0017] Erbas, M. , Candal, C. , Kilic, O. , & Mutlu, C. (2016). Determination and solution of moisture sorption isotherms of foods. GIDA /The Journal of Food, 41(3), 171–178. 10.15237/gida.gd15045

[jfds17061-bib-0018] Farinetti, A. , Zurlo, V. , Manenti, A. , Coppi, F. , & Mattioli, A. V. (2017). Mediterranean diet and colorectal cancer: A systematic review. Nutrition (Burbank, Los Angeles County, Calif.), 43–44, 83–88. 10.1016/J.NUT.2017.06.008 28935150

[jfds17061-bib-0019] Fernández‐Pan, I. , Ziani, K. , Pedroza‐Islas, R. , & Maté, J. I. (2010). Effect of drying conditions on the mechanical and barrier properties of films based on chitosan. Drying Technology, 28(12), 1350–1358. 10.1080/07373937.2010.482692

[jfds17061-bib-0020] Fiorentini, C. , Demarchi, S. M. , Quintero Ruiz, N. A. , Torrez Irigoyen, R. M. , & Giner, S. A. (2015). Arrhenius activation energy for water diffusion during drying of tomato leathers: The concept of characteristic product temperature. Biosystems Engineering, 132, 39–46. 10.1016/j.biosystemseng.2015.02.004

[jfds17061-bib-0021] Goodman, C. L. , Fawcett, S. , & Barringer, S. A. (2002). Flavor, viscosity, and color analyses of hot and cold break tomato juices. Journal of Food Science, 67(1), 404–408. 10.1111/j.1365-2621.2002.tb11418.x 25603846

[jfds17061-bib-0022] Grosso, G. , Buscemi, S. , Galvano, F. , Mistretta, A. , Marventano, S. , Vela, V. L. , Drago, F. , Gangi, S. , Basile, F. , & Biondi, A. (2013). Mediterranean diet and cancer: Epidemiological evidence and mechanism of selected aspects. BMC Surgery, 13(S2), S14. 10.1186/1471-2482-13-S2-S14 24267672 PMC3850991

[jfds17061-bib-0023] Hashemi, R. H. , Bradley, W. G. , & Lisanti, C. J. (2010). MRI : The basics. Lippincott Williams & Wilkins.

[jfds17061-bib-0024] Hazra, S. K. , Sarkar, T. , Salauddin, M. , Sheikh, H. I. , Pati, S. , & Chakraborty, R. (2020). Characterization of phytochemicals, minerals and in vitro medicinal activities of bael (*Aegle marmelos* L.) pulp and differently dried edible leathers. Heliyon, 6(10), e05382. 10.1016/j.heliyon.2020.e05382 33163665 PMC7610326

[jfds17061-bib-0025] Hepsağ, F. , & Kizildeniz, T. (2021). Pesticide residues and health risk appraisal of tomato cultivated in greenhouse from the Mediterranean region of Turkey. Environmental Science and Pollution Research, 28(18), 22551–22562. 10.1007/s11356-020-12232-7 33420929

[jfds17061-bib-0026] Huang, X. , & Hsieh, F.‐H. (2006). Physical properties, sensory attributes, and consumer preference of pear fruit leather. Journal of Food Science, 70(3), E177–E186. 10.1111/j.1365-2621.2005.tb07133.x

[jfds17061-bib-0027] Kampatsikas, I. , Bijelic, A. , & Rompel, A. (2019). Biochemical and structural characterization of tomato polyphenol oxidases provide novel insights into their substrate specificity. Scientific Reports, 9(1), 4022. 10.1038/s41598-019-39687-0 30858490 PMC6411738

[jfds17061-bib-0028] Kaya, A. , Fahrettin, G. , & Medeni, M. (2002). Moisture sorption isotherms of grape pestil and foamed grape pestil. Nahrung/Food, 46(2), 73–75. 10.1002/1521-3803(20020301)46:2<73::AID-FOOD73>3.0.CO;2-N 12017994

[jfds17061-bib-0029] Kurniadi, M. , Parnanto, N. H. R. , Saputri, M. W. , Sari, A. M. , Indrianingsih, A. W. , Herawati, E. R. N. , Ariani, D. , Juligani, B. , Kusumaningrum, A. , & Frediansyah, A. (2022). The effect of kappa‐carrageenan and gum Arabic on the production of guava‐banana fruit leather. Journal of Food Science and Technology, 59(11), 4415–4426. 10.1007/s13197-022-05521-1 35812463 PMC9253237

[jfds17061-bib-0030] Majumdar, A. , Pradhan, N. , Sadasivan, J. , Acharya, A. , Ojha, N. , Babu, S. , & Bose, S. (2018). Food degradation and foodborne diseases: A microbial approach. In Handbook of food bioengineering, microbial contamination and food degradation (pp. 109–148). Academic Press. 10.1016/B978-0-12-811515-2.00005-6

[jfds17061-bib-0031] Mariette, F. (2009). Investigations of food colloids by NMR and MRI. Current Opinion in Colloid & Interface Science, 14(3), 203–211. 10.1016/j.cocis.2008.10.006

[jfds17061-bib-0032] Maskan, A. , Kaya, S. , & Maskan, M. (2002). Hot air and sun drying of grape leather (pestil). Journal of Food Engineering, 54(1), 81–88. 10.1016/S0260-8774(01)00188-1

[jfds17061-bib-0033] Mert, B. (2012). Using high pressure microfluidization to improve physical properties and lycopene content of ketchup type products. Journal of Food Engineering, 109(3), 579–587. 10.1016/j.jfoodeng.2011.10.021

[jfds17061-bib-0034] Mession, J.‐L. , Chihi, M. L. , Sok, N. , & Saurel, R. (2015). Effect of globular pea proteins fractionation on their heat‐induced aggregation and acid cold‐set gelation. Food Hydrocolloids, 46, 233–243. 10.1016/j.foodhyd.2014.11.025

[jfds17061-bib-0035] Nandane, A. S. , & Jain, R. (2014). Study of mechanical properties of soy protein based edible film as affected by its composition and process parameters by using RSM. Journal of Food Science and Technology, 52, 3645–3650. 10.1007/s13197-014-1417-4 26028747 PMC4444901

[jfds17061-bib-0036] Nayaka, V. S. K. , Tiwari, R. B. , Narayana, C. K. , Ranjitha, K. , Azeez, S. , Vasugi, C. , Venugopalan, R. , Bhuvaneswari, S. , & Sujayasree, O. J. (2022). Comparative effect of different sugars instigating non‐enzymatic browning and Maillard reaction products in guava fruit leather. Journal of Horticultural Sciences, 17(1), 174–183. 10.24154/jhs.v17i1.1387

[jfds17061-bib-0037] Nizamlioglu, N. M. , Yasar, S. , & Bulut, Y. (2022). Chemical versus infrared spectroscopic measurements of quality attributes of sun or oven dried fruit leathers from apple, plum and apple‐plum mixture. LWT, 153, 112420. 10.1016/j.lwt.2021.112420

[jfds17061-bib-0038] Okilya, S. , Mukisa, I. M. , & Kaaya, A. N. (2010). Effect of solar drying on the quality and acceptability of jackfruit leather. Electronic Journal of Environmental, Agricultural and Food Chemistry, 9, 101–111.

[jfds17061-bib-0039] Ozel, B. , & Oztop, M. H. (2021). A quick look to the use of time domain nuclear magnetic resonance relaxometry and magnetic resonance imaging for food quality applications. Current Opinion in Food Science, 41, 122–129. 10.1016/j.cofs.2021.03.012

[jfds17061-bib-0040] Periago, M. J. , Rincón, F. , Agüera, M. D. , & Ros, G. (2004). Mixture approach for optimizing lycopene extraction from tomato and tomato products. Journal of Agricultural and Food Chemistry, 52(19), 5796–5802. 10.1021/jf049345h 15366823

[jfds17061-bib-0041] Phimpharian, C. , Jangchud, A. , Jangchud, K. , Therdthai, N. , Prinyawiwatkul, W. , & No, H. K. (2011). Physicochemical characteristics and sensory optimisation of pineapple leather snack as affected by glucose syrup and pectin concentrations. International Journal of Food Science & Technology, 46(5), 972–981. 10.1111/j.1365-2621.2011.02579.x

[jfds17061-bib-0042] Rathee, R. , & Rajain, P. (2019). Role colour plays in influencing consumer behaviour. International Research Journal of Business Studies, 12(3), 209–222.

[jfds17061-bib-0043] Roha, S. , Zainal, S. , Noriham, A. , & Nadzirah, K. Z. (2013). Determination of sugar content in pineapple waste variety N36. International Food Research Journal, 20(4), 1941–1943.

[jfds17061-bib-0044] Sahin, S. , & Sumnu, S. G. (2006). Physical properties of foods. Springer. 10.1007/0-387-30808-3

[jfds17061-bib-0045] Shi, J. , & Maguer, M. L. (2000). Lycopene in Tomatoes: Chemical and physical properties affected by food processing. Critical Reviews in Food Science and Nutrition, 40(1), 1–42. 10.1080/10408690091189275 10674200

[jfds17061-bib-0046] Singh, A. , Sonkar, C. , & Shingh, S. (2019). Studies on development of process and product of plum fruit leather. International Journal of Food Science and Nutrition, 4(5), 74–79.

[jfds17061-bib-0047] Spagna, G. , Barbagallo, R. N. , Chisari, M. , & Branca, F. (2005). Characterization of a tomato polyphenol oxidase and its role in browning and lycopene content. Journal of Agricultural and Food Chemistry, 53(6), 2032–2038. 10.1021/jf040336i 15769132

[jfds17061-bib-0048] Valenzuela, C. , & Aguilera, J. M. (2013). Aerated apple leathers: Effect of microstructure on drying and mechanical properties. Drying Technology, 31(16), 1951–1959. 10.1080/07373937.2013.803979

[jfds17061-bib-0049] Valenzuela, C. , & Aguilera, J. M. (2015). Effects of different factors on stickiness of apple leathers. Journal of Food Engineering, 149, 51–60. 10.1016/j.jfoodeng.2014.09.029

[jfds17061-bib-0050] Vatthanakul, S. , Jangchud, A. , Jangchud, K. , Therdthai, N. , & Wilkinson, B. (2010). Gold kiwifruit leather product development using quality function deployment approach. Food Quality and Preference, 21(3), 339–345. 10.1016/j.foodqual.2009.06.002

[jfds17061-bib-0051] Yılmaz, F. M. , Yüksekkaya, S. , Vardin, H. , & Karaaslan, M. (2017). The effects of drying conditions on moisture transfer and quality of pomegranate fruit leather (pestil). Journal of the Saudi Society of Agricultural Sciences, 16(1), 33–40.

[jfds17061-bib-0052] Zechmeister, L. , LeRosen, L. , Schroeder, W. A. , Polgar, A. , & Pauling, L. (1943). Spectral characteristics and configuration of some stereoisomeric carotenoids including prolycopene and pro‐y‐carotene. Journal of the American Chemical Society, 65(10), 1940–1951. 10.1021/ja01250a039

